# The genetic heist: how transposable elements capture and repurpose genes in plants

**DOI:** 10.1186/s13059-026-04054-6

**Published:** 2026-05-07

**Authors:** Navei Cerda-Hernandez, Thomas Bureau

**Affiliations:** McGill University, Montreal, Canada

## Abstract

Transposable elements are pervasive components of plant genomes, and their role as dynamic architects of genome evolution is increasingly recognized. Beyond their ability to mobilize within genomes, transposable elements facilitate the capture and propagation of host-derived sequences, profoundly influencing genome plasticity. In this review, we provide a comprehensive synthesis of mechanisms of gene capture. These processes can contribute to novel gene formation, gene duplication, and the modification of gene expression regulation, ultimately driving transcriptomic and phenotypic diversity. This review lays the groundwork for crucial future research exploring the evolutionary and functional significance of transposable element-mediated gene capture in plants.

## Introduction

Transposable elements (TEs) are major architects of plant genome structure, evolution and plasticity. Making up large fractions of many plant genomes, TEs can influence gene expression, chromatin organization, and genome size dynamics [1, 2]. Since McClintock’s pioneering work in maize demonstrated that TEs can alter gene activity and genomic structure [3], studies across diverse plant lineages have shown that TE mobility and accumulation have contributed extensively to genome diversification in angiosperms and other plant groups [4, 5].

TEs are broadly divided into two major classes based on their mobilization mechanisms. Class I elements, or retrotransposons (RTs), transpose through an RNA intermediate that is reverse transcribed and inserted elsewhere in the genome in a “copy-and-paste” process that leaves the original element intact. Class II elements (DNA TEs) typically move via excision and reintegration in a “cut-and-paste” mechanism [6], or in some cases through replication of a circular DNA intermediate in a “peel-and-paste” pathway [7]. Each class can be subdivided into orders defined by their enzymatic machinery, integration strategy and structure of the TE: Class I includes LTR RTs, LINEs, SINEs, PLEs and DIRS, whereas Class II includes TIR TEs, Helitrons, Cryptons and Mavericks (the latter two not described in plants) [8]. TEs may be autonomous, encoding the proteins necessary for their own mobilisation, or non-autonomous, relying on enzymatic machinery supplied by related autonomous elements.

In addition to their well-documented regulatory and structural effects, TEs can sometimes capture host genome sequences (often gene fragments), mobilize, and amplify them together with their own sequence in a process referred to as gene capture [7, 9]. This phenomenon of gene capture by TEs can have a profound evolutionary effect. By reshuffling and amplifying gene fragments, TEs have been implicated in modifying gene expression patterns and introducing new regulatory interactions [10, 11], the insertion of genes into a new genomic location through reverse transcription [12, 13], and in some cases the incorporation of a captured exon into the host transcripts through alternative splicing and the production of a new protein that shows signatures of selection [4, 14, 15].

Highly successful mobilisation of TEs presents both a challenge and a source of innovation for their host genome. Plants rely on multilayered silencing mechanisms, such as RNA-guided repression and epigenetic modification, to constrain TE activity [16, 17], yet their chromosomal distribution remains influenced by insertion site preferences [18], natural selection, and drift [8]. TE lineages can colonize distinct genomic niches, from gene-rich euchromatin [8] to pericentromeric regions enriched in tandem repeats and recent TE insertions [19, 20], while other TEs undergo “local hopping” that keeps them in proximity to their donor loci [21]. These spatial biases not only influence genome architecture but also create opportunities for physical interactions between TEs and adjacent genes, setting the stage for gene fragment acquisition and mobilisation.

Despite significant advances, many questions remain about the mechanisms that govern the retention and functional integration of captured gene sequences in plant genomes. Distinguishing between the neutral and adaptive impacts of gene capture poses a substantial challenge [22]. Moreover, the extent to which captured genes are retained and functionally integrated into host genomes, the regulatory dynamics they introduce, and their long-term evolutionary fate are still subjects of active investigation [5].

Given the abundance of captured gene fragments and the growing recognition of their evolutionary significance, understanding how TEs capture and mobilize host genome sequences is essential for interpreting their functional consequences. Recent syntheses have focused largely on animal systems or have discussed plants only briefly, leaving plant-specific insights scattered across a wide array of genomic analyses. Therefore, the aim of this review is to consolidate and critically evaluate a comprehensive overview of the mechanisms by which TEs capture host genes, the evolutionary significance of this process, and the emerging insights from recent genomic studies in plants. By integrating findings from diverse plant organisms, we highlight the role of TEs as powerful agents of genome evolution and explore potential avenues for future research in this rapidly evolving field.

## Mechanisms of gene capture by TEs

### Transduction

**Fig. 1:**
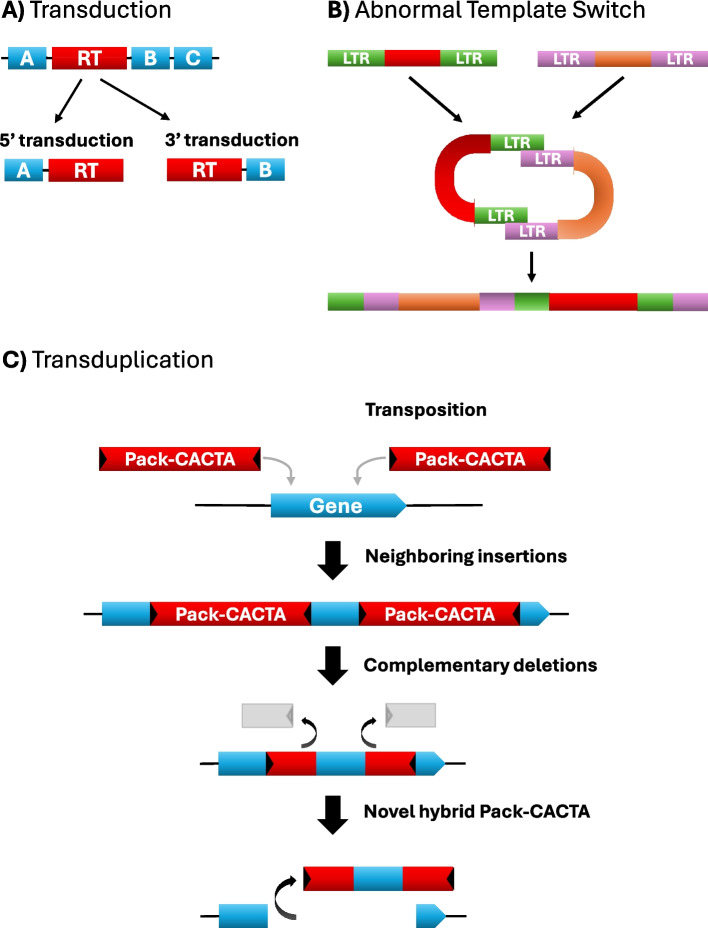
Mechanisms of gene capture. **A** In transduction, transcription read-through of a RT can lead to upstream (left) or downstream (right) flanking genomic sequences to be mobilized along with the RT. **B** On abnormal template switch, a template switch complex is formed by the inter-strand pairing of compatible RNAs from 2 different RTs, resulting in an insertion that has homology between the 2 external RTs. **C** Transduplication by Pack-CACTA TEs has been proposed to occur when new TE insertions form a closely spaced tandem array. The neighbouring TEs go through a process of aberrant excisions of complementary, terminal deletions, resulting in a novel hybrid Pack-CACTA TE with an integrated genomic sequence. Red and orange blocks represent TE sequences. Pink and Green blocks represent the LTR regions of a RT. The black triangles represent the TIRs of a DNA TE. Blue blocks represent host genes, and black lines represent genomic sequences. Panels B) and C) are adapted from the original figures presented in [39] and [21], respectively

TEs, particularly RTs, can sometimes carry flanking host genome sequences as a result of transcription read-through [23, 24]. This phenomenon can involve genomic sequences upstream or downstream of the RT, termed 5’- and 3’-transduction respectively (Fig. 1-A). In 3’-transduction, a RT with a weak polyadenylation signal causes the RNA transcription machinery to skip the terminus of the RT, and continue transcription until it finds an alternative polyadenylation signal located downstream in the 3’ adjacent genomic sequence [25]. Similarly, 5’-transduction occurs when transcription is initiated via a promoter upstream of the RT and the transcript is extended to include sequences leading into the RT [26]. Gene transduction results in a chimeric transcript that contains both RT-derived sequences and adjacent host genomic sequences [27]. The captured sequences are usually called transduced segments, and can have regulatory elements, exons, or even full-length genes, with the potential to alter their expression or function after reinsertion into the genome [28, 29].

One of the first examples of host DNA captured by a plant RT was the *Bs1* RT in maize, where domains of the host plasma membrane ATPase gene were found to be encoded within the *Bs1* element sequence [30, 31]. Briefly, the *Bs1* RT of maize was reported to have undergone 3 distinct transductions of segments from three different host genes (proton-dependent membrane ATPase, xylan endohydrolase, and β−1,3-glucanase). Although none of the transduced segments had full-length ORFs, subsequent mutations and exon shuffling events allowed the creation of a new chimeric gene that is both transcribed and translated and is potentially involved in reproductive development [32, 33]. Additionally, in *A. thaliana*, *3’-*transduction of a fragment of a gene encoding a cytochrome P450-like protein was described in a group of LTR RT called Terminal-repeat retrotransposons in miniature (*TRIM*) [34]. Currently, the only experimentally characterized transduced segment in plants has been done for a RT called *Rider* in tomato plants. Compared with the ancestral copy, the *Rider* RT carries a transduced segment of the *sun* locus and shows increased expression, which results in morphologically different tomatoes with an elongated fruit shape [35].

According to studies in mammalian genomes, 5’-transduction is less common but equally impactful [36]. In the human genome, a subfamily of *SVA* RTs exhibit a high number of 5’-transductions of host genomic sequences as a result of *SVA* retrotransposons frequently parasitizing nearby host gene promoters [26, 37]. However, examples of 5’-transduction in plants have not been reported.

### Abnormal template switching

Typically, the LTR regions of LTR RTs contain three regions: i) the U3 (unique 3’) region, ii) the R (redundant or repeated) region, and iii) the U5 (unique 5’) region. Immediately before reverse transcription, two RNA molecules from the LTR RT are packaged into a virus-like structure called the nucleocapsid and the RNA matrix forms a loop via the high homology between R regions (both the 5’ and 3’ R regions in the LTRs) of the RNA molecules as a guide [38]. This buckle allows the synthesis of cDNA to proceed upon reaching the 5’ end of the template by ‘jumping’ across to the 3’ end of the template and continuing reverse transcription, a process known as template switching. This process results in perfect identity between the 5’ and 3’ LTRs of the newly synthesized RT [39].

During the reverse transcription step, an ‘accident’ can occur during the template switching stage, where the switch occurs between two unrelated RNAs packaged in the same nucleocapsid, leading to the formation of chimeric products that introduce novel sequences into a new copy of a RT (Fig. 1-B) [40]. This has been observed in the *Veju TRIM* elements, where an unknown DNA sequence was introduced and formed a longer version of *Veju TRIMs* that have differentially evolved in wheat genomes [41]. An additional example lies in the *BARE-1* family of RTs from barley, where abnormal template switching led to the introduction of a *Wis-2* RT sequence into the LTR region of a subgroup of *BARE-1* RTs which gave rise to a new promoter class called *BARE-2* [42].

### Transduplication

Recently, a study in *A. thaliana* and rice revealed a mechanism by which *Pack*-Type TIR DNA TEs can capture genomic sequences [21]. In this case, related *Pack-CACTA* TEs in *A. thaliana* can have new insertions in a closely spaced tandem array. These neighbouring elements subsequently undergo a process of aberrant excision that ends with complementary, terminal deletions of the 3’-end in one TE and the 5’-end of the neighbouring TE. This process results in a hybrid *Pack*-Type TE that incorporates chromosomal DNA within the new boundaries of the hybrid element (Fig. 1-C), and is therefore able to mobilize the genomic sequence captured in a subsequent transposition event [21]. However, there still might be an alternative, yet undiscovered mechanism, by which DNA TEs can capture and integrate fragments of unlinked cellular genes between the termini of the TE. These mechanisms of gene capture by DNA TEs are known as transduplication [43]. The gene fragments captured are usually non-functional pseudogenes, although numerous fragments have been found to be actively expressed and exhibit indications of selection, suggesting some level of functionality [14]. For example, over 3,000 *Pack-MULE* TEs in rice captured and mobilize fragments derived from more than 1,000 host genes, some of which are expressed as chimeric transcripts [44]. Similarly, approximately 2,791 transcriptionally active Helitron TEs carrying captured sequences derived from 376 different host genes have been reported in maize [45, 46].

In contrast, some cases in which a transduplicated exon is incorporated into the host transcripts through alternative splicing and produces a new protein have been reported [15]. In *A. thaliana,* a group of Non-TIR MULE DNA TEs were found to contain a transduplicated Ulp1 domain [47], which is an important domain from an enzyme involved in different plant signalling pathways. The genes carrying the transduplicated Ulp1 domain are called *Kaonashi* (KIU), and represent one, out of three, coding genes inside this group of Non-TIR MULEs [47]. Nevertheless, the putative new protein has not been thoroughly characterized. Similar transduplicated Ulp1 sequences like KIU have also been described in MULE TEs of grapevine [48], melon, and rice [49].

## Evolutionary impact of gene capture by TEs

### Novel gene formation and neofunctionalization

As previously mentioned, the most common evolutionary fate of captured gene sequences is pseudogenization or gene loss. However, it is possible for TE captured gene sequences to maintain their transcription potential and encode new proteins that can evolve through various routes, including neofunctionalization, regulatory innovation, and positive dosage [50].

A recent systematic study of *Pack*-Type TEs in rice and maize revealed how insertions of different *Pack*-Type TEs nearby or within genes in syntenic chromosomal regions between 4 Poaceae plants (maize, sorghum, foxtail millet and stiff brome) give rise to new functional proteins by introducing transduplicated sequences into the transcripts via the exon-shuffling mechanism [4]. Using an enrichment of GO terms, researchers reported that sequences captured by *Pack*-Type TEs in both rice and maize were significantly enriched in categories such as post-embryonic development, flower development, oxidative stress, regulation of transcription, and protein localization, and argued that by introducing these captured sequences to the transcripts of host genes, *Pack*-Type TEs have had a strong impact on plant genome evolution [4].

Additionally in rice, around 1,235 genes derived from captured LTR sequences have been identified, and substitution analysis revealed that most of these genes are under negative selection, suggesting continued function [51]. In sorghum, 1343 transduced sequences were identified in LTR retrotransposons of which up to 72.4% of the captured sequences were expressed and showed evidence of positive selection, suggesting that transduced genes might have evolved novel gene functions [52], further showing how captured gene sequences can sometimes be retained by the host.

### Transcriptional regulatory innovation

TEs frequently contribute cis-regulatory sequences to plant genomes, including full promoters, enhancers and short transcription factor binding sites (TFBS), and in doing so they can rewire transcriptional networks [11]. For instance, Miniature Inverted-repeat Transposable Elements (*MITEs*) in *A. thaliana* captured a binding motif for the E2F family of transcription factors, which is important for the regulation of various cell cycle related processes [53]. The capture of E2F motifs by *MITEs* likely occurred early in the Brassicaceae clade and was then differentially amplified during Brassicaceae evolution; in *A. thaliana* some insertions of the E2F-containing *MITEs* landed in close proximity to promoters and can influence the regulatory programs of neighbouring genes [53, 54]. In maize, genome-wide surveys have shown that a subset of TEs have the potential to capture and carry sequences that facilitate an accessible chromatin state and can explain expression variation of nearby genes across genotypes, suggesting that TE with presence/absence of accessible chromatin regions can directly contribute to regulatory variation relevant for domestication and adaptation [10].

A comprehensive study in Brassicas showed that *MITE* DNA TEs are significantly enriched for functional TFBS and preferentially insert into gene-proximal regions, effectively acting as mobile regulatory cassettes. Their widespread motif redistribution has the potential to rewire transcriptional networks by altering the regulatory connectivity of genes that are responsible for differential adaptive responses and breeding traits in plants, like response to stress, flowering time, or fruit ripening [55]. In *A.thaliana* binding sites for PHE1, a key TF involved in the regulation of paternally expressed imprinted genes, was shown to have been spread by RC/Helitron TEs that captured and amplified the TFBS throughout *A.thaliana*’s genome [56]. Other notable examples of captured TFBS are the bZIP60 and PIF3 TFs in peach and Japanese plum, which are key regulators of pathways related to biotic and abiotic stress responses and bud dormancy, and the TCP15/23 TFBS in tomato, involved in the fruit ripening transcriptional network [55]. Although this study does not experimentally dissect how individual MITE-borne TFBS reshape transcriptional circuits, their findings establish a critical foundation that TE-driven proliferation of TFBS is a widespread and evolutionarily consequential phenomenon in plants.

Long-read direct RNA sequencing has further revealed that TE-derived sequences are frequently co-transcribed with genes, producing TE-gene chimeric transcript isoforms whose production and regulation are linked to specific epigenetic states and environmental responses, providing a molecular route by which TE sequences can introduce new transcription factor binding sites that can be incorporated into endogenous regulatory programs [57]. In wheat, recent work identified a large proportion of enhancer-like elements derived from TEs that are enriched in regulatory signatures and likely contribute to subgenomic differentiation of gene expression programs and polyploid developmental plasticity [58]. Similarly, a study on *Brassica napus* showed that Helitron-captured gene sequences can alter gene expression regulation by directing epigenetic modifications, and influence the establishment of subgenome dominance by synergistic regulation of siRNA mediated DNA methylation, revealing an important role of Helitron-captured sequences during the process of allopolyploidization [59].

### Epigenetic regulation

In *A. thaliana*, a family of non-TIR MULE DNA TEs, denominated *Vandal*, was found to have a gene, called *VANC*, which encodes a protein that can direct specific hypomethylation of related TEs by recognizing short sequence motifs within non-coding regions of other related *Vandal* TEs [60, 61]. Some *Vandal* TEs carry a copy of the *VANC* gene that contains a transduplicated Ulp1 domain. As previously mentioned, this group of transduplicated Ulp1-carrying *VANC* genes is known as KIUs [47]. Similar to other *Vandal* TEs, KIU-carrying *Vandals* can also recognize specific motifs in non-coding regions exclusively in other KIU-carrying *Vandals*, inducing the hypomethylation of those related TEs, allowing them to escape epigenetic silencing and thus, facilitating their mobilization [61]. The capture of the Ulp1 domain in non-TIR MULEs was found to have occurred at the separation point between monocots and eudicots, and although the precise function of the Ulp1 domain in these TEs is unknown, its’ strong conservation in the eudicot clade suggests an advantageous function [61].

Paradoxically, captured gene sequences can also lead to epigenetic silencing, as opposed to what was observed for KIU-carrying *Vandal* TEs. A systematic analysis of the maize genome, revealed that Helitrons*, Pack-MULEs,* and *Sirevirus* LTR RT, which carry captured genomic sequences from 1629 donor genes, act as siRNA reservoirs that can induce epigenetic silencing of both related TEs and the donor gene [62]. However, the degree of methylation and epigenetic silencing varies according to the putative functional importance of the donor gene. If a donor gene is essential, individuals affected by captured sequence siRNA silencing may be removed from the population by purifying selection to avoid harmful expression changes to that gene. This creates an intragenomic conflict, where the pressure to suppress the TE is counterbalanced by the potential harm of disrupting the donor gene [62]. In this way, capturing important genomic sequences could be beneficial for a TE as a way of “camouflaging” and escaping the host epigenetic silencing machinery [63].

## Conclusions and perspectives

Gene capture by TEs emerges from this review as a multifaceted evolutionary force in plants, since it can lead to reshaping gene structure, transcript diversity, contrasting regulatory landscapes, and epigenetic states. The capture of important genomic loci can lead to fruit morphological changes, as is the case for the *sun* locus transduced by RTs in tomato [35]. It can introduce extensive transcript variations in host genes, which allow the production of novel proteins that allow for the evolution and differentiation of different plants, as has been observed within the Poaceae family of plants [4]. It can change the transcription regulation landscape of genes close to a TE carrying captured gene segments [53, 54]. Alternatively, TEs can acquire sequences that modify their epigenetic regulation [61, 62]. These well characterized cases demonstrate the creative potential of captured sequences to contribute to phenotypic innovation, developmental modulation, and stress adaptation. The cumulative evidence across plant clades makes it clear that TE-mediated gene capture is not an incidental phenomenon but a recurrent feature of plant genome evolution. Yet, despite the wealth of computational and transcriptomic analyses revealing thousands of candidates captured sequences, experimental validation remains strikingly rare, leaving major gaps in our understanding of how these sequences are retained, regulated, and functionally integrated.

Looking forward, the field is now positioned for a decisive shift from cataloguing to mechanistic dissection. Several concrete experimental frameworks can accelerate progress. CRISPR/Cas9 genome editing offers a powerful avenue for testing functional hypotheses like targeted deletion of transduced segments, restoration of ancestral alleles, or promoter swaps can test whether captured sequences alter gene expression, protein function, or physiological traits. A growing number of studies now illustrate that cis-regulatory editing via CRISPR/Cas9 is feasible in plants and capable of generating heritable, trait-altering variation [64, 65]. Recent innovations even combine CRISPR with transposase systems like the *Pong* transposase in rice to enable programmable insertion of regulatory modules into plant genomes [66]. Precise deletion of *Tos17* RT insertions via CRISPR/Cas9-mediated genome editing has been demonstrated in rice [67], underscoring the practicability of ancestral allele restoration. Additionally, editing of TE termini or transposase domains in active elements could illuminate the mechanistic basis of capture pathways with experimentally tractable mobilisation dynamics, particularly for the wide collection of *Pack*-Type TEs reported in *A. thaliana*, rice, and maize [4, 21], the *Bs1* RTs in maize [30, 32, 33], the *Veju TRIMs* in wheat [39, 41], or the *BARE-2* in barley [42].

As previously mentioned, recent work using long-read direct RNA sequencing in *A.thaliana* demonstrated that TE-gene chimeric transcripts are widespread and epigenetically regulated [57], illustrating the power of full-length transcriptomics to reveal TE-derived regulatory architecture. Meanwhile, a comprehensive review of plant single-cell and spatial omics argue that these approaches, though underused, provide unique opportunities to link TE-derived promoters, enhancers and TFBS to cell-type or tissue-specific expression programs [68]. Together, these emerging tools offer a promising path toward understanding how TEs reshape regulatory networks in a more specific spatial and cellular context. Epigenomic profiling techniques, such as whole-genome bisulfite sequencing, ChIP-seq and ATAC-seq, can be utilized in TE mutant lines to further examine how captured sequences influence the accessible chromatin landscape, subgenome dominance, and dosage regulation, reducing the reliance on bulk data inference. Finally, comparative and population genomics studies across diverse plant species can help distinguish adaptive from neutral outcomes by identifying recurrently retained captured sequences, signatures of selection, and associations with ecologically or agriculturally relevant traits. For example, a recent pangenome analysis of TE insertion polymorphisms in rice revealed over 30,000 polymorphic TE insertions across 165 rice accessions that can influence epigenetic differentiation under cold stress [69].

Hopefully, the approaches discussed on this review will allow researchers to move beyond descriptive models and directly test functional, regulatory, and evolutionary consequences of TE-mediated gene capture. Plant genomes offer unique experimental advantages, including active TE families, rich phenotypic diversity, and extensive genomic resources, positioning them as ideal systems in which to uncover how TEs generate new genetic material and modulate adaptability through mechanisms like gene capture. A deeper mechanistic understanding of these processes promises not only to shine a light into the evolutionary dynamics of plant genomes, but also to reveal opportunities for leveraging TE-mediated innovation in crop improvement and synthetic biology.

## Data Availability

No datasets were generated or analysed during the current study.
